# High-throughput identification of post-transcriptional utrophin up-regulators for Duchenne muscle dystrophy (DMD) therapy

**DOI:** 10.1038/s41598-020-58737-6

**Published:** 2020-02-07

**Authors:** Emanuele Loro, Kasturi Sengupta, Sasha Bogdanovich, Kanupriya Whig, David C. Schultz, Donna M. Huryn, Tejvir S. Khurana

**Affiliations:** 1 Department of Physiology and Pennsylvania Muscle Institute, Perelman School of Medicine, University of Pennsylvania, Philadelphia, PA USA; 2 High-Throughput Screening Core, University of Pennsylvania, Philadelphia, PA USA; 3 Department of Pharmaceutical Sciences, University of Pittsburgh, Pittsburgh, PA USA

**Keywords:** Target identification, Neuromuscular disease

## Abstract

Upregulation of endogenous utrophin offers great promise for treating DMD, as it can functionally compensate for the lack of dystrophin caused by DMD gene mutations, without the immunogenic concerns associated with delivering dystrophin. However, post-transcriptional repression mechanisms targeting the 5′ and 3′ untranslated regions (UTRs) of utrophin mRNA significantly limit the magnitude of utrophin upregulation achievable by promoter activation. Using a utrophin 5′3′UTR reporter assay, we performed a high-throughput screen (HTS) for small molecules capable of relieving utrophin post-transcriptional repression. We identified 27 hits that were ranked using a using an algorithm that we designed for hit prioritization that we call Hit to Lead Prioritization Score (H2LPS). The top 10 hits were validated using an orthogonal assay for endogenous utrophin expression. Evaluation of the top scoring hit, Trichostatin A (TSA), demonstrated utrophin upregulation and functional improvement in the *mdx* mouse model of DMD. TSA and the other small molecules identified here represent potential starting points for DMD drug discovery efforts.

## Introduction

DMD is a devastating X-linked neuromuscular disorder that effects ca. 1 in 3500 live males worldwide. DMD is caused by mutations in the DMD gene resulting in the loss or severe reduction of dystrophin protein expression^1,2^. Dystrophin is thought to provide structural support to muscle fibers by linking the sub-sarcolemmal actin cytoskeleton to the extracellular matrix via the dystrophin-associated glycoprotein complex. In the absence of dystrophin, the complex is lost from the sarcolemma and the myofiber becomes susceptible to damage during contraction-relaxation cycles. Increased damage leads to chronic inflammation and progressive replacement of contractile units with fibro-fatty tissue, contributing to significant muscle wasting^3^. DMD patients are typically diagnosed in early childhood and become increasingly wheelchair dependent in their teens, with cardiac and respiratory failure being the major causes of morbidity and mortality^4–6^. There is currently no definitive cure for the disease.

Corticosteroids (e.g. prednisone) have been in use for over 50 years for DMD and, while not specific as a therapeutic for DMD, can slow the progression of the disease^7–10^. Indeed, with early intervention, they have been shown to increase life span of patients^11^. However, their use is associated with extensive side effects and toxicities exemplified by immune suppression, osteoporosis, and weight gain. Newer steroid drugs such as vamorolone^12^ and deflazacort^13^ are promising candidates that are suggested to retain beneficial effects of prednisone without many of the side effects. DMD-specific approaches, including viral gene therapy which aims to reintroduce shorter functional versions of the dystrophin gene^14^, or exon skipping which utilizes stable oligonucleotides to skip one or more exons in order to regain expression of shorter functional dystrophin^15,16^, are currently in various stages of clinical evaluation. Because the resulting version of dystrophin transcript will be shorter than normal, however, these therapies would be predicted to only decrease the severity of the disease (*i.e.* convert from DMD to a milder Becker allelic form). Other approaches currently under development include promoting the read-through of premature stop codons and correcting mutations using gene editing^17,18^. However, all these approaches are mutation-specific and therefore would be applicable to restricted subsets of DMD patients.

An alternate strategy for a DMD-specific therapy that in principle would be applicable to all patients would be to increase the expression of the autosomal-encoded dystrophin-related protein homolog, utrophin^19,20^. Like dystrophin, utrophin (also known as dystrophin-related protein-DRP) is a member of the spectrin superfamily and shares extensive sequence similarity and functional motifs with dystrophin, including the capacity to bind the same dystrophin associated glycoprotein complex. Utrophin is expressed at high levels in fetal tissue and developmentally downregulated in adults. In the *mdx* mouse model of DMD the developmentally-regulated decline in utrophin levels corresponds with the onset of muscle necrosis^21^. Expression of truncated^22–24^ or full-length^25^ utrophin significantly ameliorates the phenotype of *mdx* mice and provides the rationale for harnessing pharmacological upregulation of endogenous utrophin as a therapeutic strategy for DMD.

The molecules and mechanisms regulating utrophin expression have been the subject of detailed mapping and characterization, in part to determine mechanisms that could be targeted to drive utrophin upregulation for DMD^26^. Utrophin has a broad tissue distribution and has a number of isoforms driven by distinct promoters^27–30^. The Utrophin A isoform is the predominant isoform expressed in muscle and hence has been the subject of concerted studies by our group and others that have mapped the major regulatory motifs and validated trans-activating and repressing factors^26,31^. Unfortunately, despite these intense efforts, none are currently clinically applicable because of the limited magnitude of upregulation achieved so far by targeting the promoter^32–34^. This has been recognized to be, at least in part, due to the fact that regulation of utrophin expression is more complex than previously appreciated^35–40^. Hence, promoter trans-activating molecules may not suffice for therapeutics by themselves. Detailed molecular analyses of utrophin mRNA and protein expression have demonstrated that utrophin is subject to significant post-transcriptional regulation, as exemplified by the transcription-translation mismatch in developing muscle cells, in different muscle groups as well as in the CNS^28,38,41^. Importantly, a variety of mechanisms targeting the 5′ and 3′UTRs of the utrophin mRNA significantly contribute to repressing utrophin protein expression in adult muscle. The 5′UTR contains a putative IRES site and been shown to be important for regulation of utrophin protein levels during regeneration and in response to steroids. Additionally, two cis-acting elements, along with a short uORF, have been found in the 5′UTR and have been suggested to repress cap dependent translation^42^. The 3′UTR contains a series of conserved AU-rich elements (AREs) as well as multiple miRNA binding sites that provide an additional layer of regulation^35–39^. We and others have shown that the interaction between utrophin mRNA and miRNAs can be targeted *in vitro* and *in vivo*^36,43–45^ to upregulate utrophin and ameliorate the dystrophic phenotype. Together, these studies provide a strong rationale for identifying small molecules capable of interacting with the 5′ and/or 3′UTR to upregulate utrophin expression. Toward this end, we have developed a stable reporter cell line containing the luciferase gene flanked by the 5′ and 3′UTR regions of the human utrophin gene^46^. In the current study, we have used this assay together with a counterassay to screen a library of 3127 small molecules including c. 1000 FDA approved drugs.

We also developed and applied a two-step pipeline which uses cluster analysis to group molecules with similar activity profiles, and then ranks them according to an automated *Hit* 2 *Lead Performance Score* (H2LPS) algorithm based on efficacy, potency and specificity. We ranked molecules according to the H2LPS and validated the top 10 using orthogonal assays for endogenous utrophin expression *in vitro*. Evaluation of the top scoring hit in the *mdx* mouse model of DMD increased utrophin expression and resulted in functional improvement of the dystrophic phenotype. Our study validates our screening assay paradigm for post-transcriptional utrophin up-regulators, including the application of an automated scoring methodology which can be applied to larger compound libraries thereby potentially identifying additional novel starting points for DMD therapeutic development.

## Results

### Implementation of the cell-based high-throughput screening

**Figure 1:**
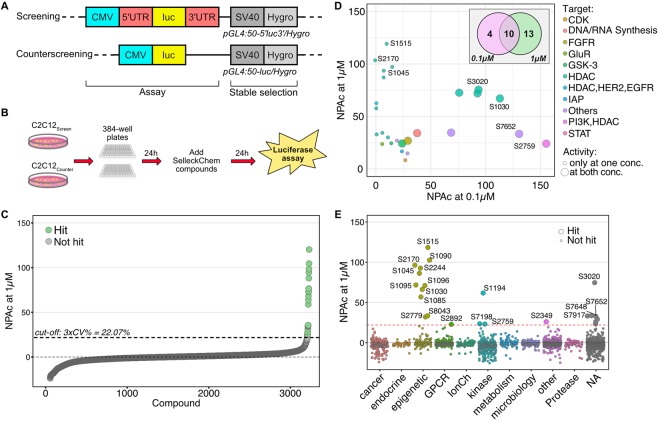
High-Throughput Screening implementation. (**A**) Diagram of the screening and counterscreening constructs used to generate the stable C_2_C_12_-based assays. (**B**) Experimental strategy: cells were plated in 384-well plates and allowed to attach for 24 hours, then treated with compounds and incubated for 24 hours before assaying luciferase activity. (**C**) Scatter plot of Normalized Percent Activation (NPAc = (DMSO_avg_ − Test well)/(DMSO_avg_ − Panobinostat_avg_) × 100) for each compound, assayed at 1 µM concentrations. NPAc was used instead of Z-scores when comparing different conditions (e.g. dosages) for the same assay. (**D**) Scatterplot comparing the NPAc at 0.1 µM and 1 µM for the 27 hits. Large circles indicate molecules active at both concentrations. Inset: Venn diagram of the number of hits identified at the two concentrations tested. (**E**) Scatter plot of NPAc for the different compounds assayed at 1 µM concentrations, organized into different target classes.

The construct for generating the screening C_2_C_12_ cell line carrying the 5′ and 3′UTR of the human utrophin gene was previously described by our group^46^. We re-derived the screening and counterscreening stable lines in early passage C_2_C_12_ cells (Fig. 1A), testing for the incorporation and functionality of the reporter transgene. Some compounds could increase light emission independently from the 5′ and 3′UTR regions, i.e. by activating the CMV promoter or increasing overall mRNA synthesis. Therefore, in order to distinguish such compounds, we generated a counterscreening cell line stably expressing only the CMV-driven luciferase gene (Fig. 1A). To determine the optimal conditions for implementation in a 384-wells high-throughput format, we evaluated DMSO tolerance, optimal cell density and incubation time (Suppl. Fig. 1A–C). The screening line performed robustly at all the tested concentrations of DMSO (0 to 1%), with coefficient of variation (CV) lower than 10% for both luciferase activity and cell viability (quantified as ATP content) (Table 1 and Suppl. Fig. 1A). Luciferase activity (Suppl. Fig. 1B) and cell viability (Suppl. Fig. 1C) increased linearly as a function of cell density (500, 1000, 2000 cells/well) and incubation time (24 or 48 hours post-treatment). Based on these results, the experimental design consisted in plating 1000 cells/well, allowing them to attach for 24 h, then treating them with compounds with a 0.2% DMSO final concentration, and assaying luciferase activity after a 24 h incubation (Fig. 1B). Panobinostat (S1030) was chosen as a positive control for its capacity to induce overall mRNA transcription and luciferase expression at concentrations within the nanomolar to micromolar range in both screening and counterscreening assays (Suppl. Fig. 1D). While maximum luciferase activity was comparable in both screening and counterscreening assays after treatment with panobinostat, basal activity was approximately 80 times higher in the screening cell line (Suppl. Fig. 1D).

**Table 1:** Screening technical data.

	Selleckchem screening @0.1 µM	Selleckchem screening @1 µM	Selleckchem dose-response screening
# plates	10	10	1
# compounds tested	3127	3127	27
S/B CV% between plates	7.10	2.83	0.68
Z’ Factor	0.63	0.69	0.75
CV% negative	7.9	10.51	7.96
CV% positive	6.35	7.05	5.29
Hit-rate	0.90%		48%

### SelleckChem library screening

To identify small molecules capable of increasing utrophin expression, we screened a curated Bioactive Screening Library (SelleckChem) containing 3127 compounds with known biological and pharmacological activities, including many FDA-approved drugs. We performed the screen at two concentrations, 1 µM (Fig. 1C) and 0.1 µM (Suppl. Fig. 1E). By using a threshold of 22.07% activation, corresponding to 3 times the standard deviation of the average NPAc (Normalized Percent Activation), we identified a total of 27 hits that were active across the two concentrations. Of these, 4 were active only at 0.1 µM, suggesting possible cytotoxicity at higher concentrations, while 13 were active only at 1 µM (Fig. 1D). The majority of the hits were compounds targeting epigenetic regulators (i.e. HDACs) and kinases (i.e. PI3K, GSK-3) (Fig. 1E and Suppl. Fig. 1F). Panobinostat (S1030) was included in the screening library and, as a validation of our approach, was detected in the assay as active at both concentrations.

### Dose-response in screening and counterscreening assays

**Figure 2:**
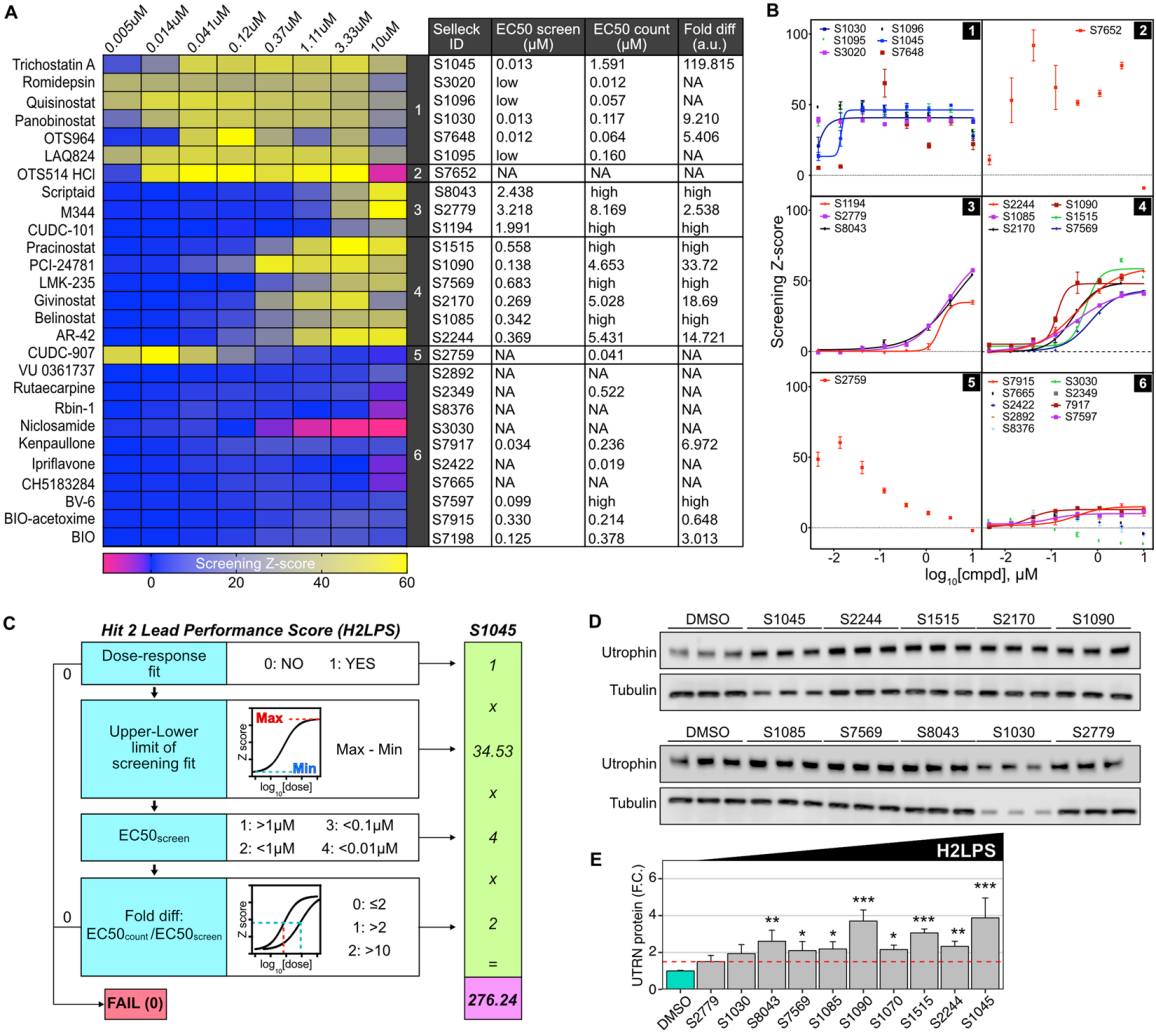
Dose-response assay, H2LPS calculation and hit validation. (**A**) Heatmap representation of dose-response traces for the 27 hit molecules. Clustering over rows was obtained by complete linkage with euclidean distance measurements. The table summarizes the EC50 values calculated from the modeled logistic 4-parameter dose-response fits, as well as the ratio between EC50 screen and EC50 counterscreen (Fold diff.). (**B**) Fitted dose-response curves organized by clusters. (**C**) Pipeline for the calculation of the H2LPS, with the example of TSA (S1045). (**D**) Representative utrophin western-blot validation in C_2_C_12_ cells of the molecules with the top 10 H2LPS, administered at 1 µM for 24 hours. The experiment was repeated 3 independent times. (**E**) Quantification of average utrophin protein levels from western-blot validation. Values are mean and standard error of the mean of three independent experiments with three replicates for each molecule. *p < 0.05; **p < 0.01; ***p < 0.001.

A first criterion for evaluating the performance of the selected hits was whether their activity was dose-dependent. After performing a dose-response analysis (5 nM to 10 µM) of each hit in the screening cell line, we applied a clustering approach (complete linkage with euclidean distance measurement) to highlight similarities between compounds profiles. The algorithm grouped the 27 compounds in 6 clusters based on their dose-response profile (Fig. 2A). From this initial analysis, cluster 2, 5 and 6 included compounds with either unsatisfactory dose-response profile, very low potency and/or with potential cytotoxicity. Clusters 1, 3 and 4 included promising compounds, with high activity and low cytotoxicity. By modeling each dose-response trace with a 4-parameter logistic model applied to the Z-score values (Fig. 2B), we calculated the EC50 of each molecule. Clusters 1 and 4 included compounds with high potency and EC50 in the order of 10 nM and 100–700 nM respectively (Fig. 2A and panels 1 and 4 of Fig. 2B). The hits in cluster 3 gave a clear dose-dependent activation but had EC50s in the µM range (Fig. 2A and panel 3 in Fig. 2B). The same 4-parameter logistic model was applied to determine whether compounds had unwanted dose-dependent activity in the counterscreening assay, likely due to a capacity to activate the CMV promoter in the reporter transgene or to increase overall mRNA transcription. From a comparison of screening and counterscreening traces, hits S1030, S3020, S2759 and S7648 had significant luciferase activity in the counterscreening assay (Suppl. Fig. 2).

### H2LPS-based ranking and hit confirmation

To automate the ranking and prioritization of hits in an objective manner for further characterization, we computed the H2LPS of individual hits, using our custom designed algorithm which takes into account the difference between EC50 determined in screening and counterscreening, as well as the dose-dependent behavior and amplitude of each fitted dose-response curve. Unacceptable values for one or more of these parameters (e.g. no dose-dependence, or similar EC50 between screening and counterscreening) cause the H2LPS to be equal to 0 (Fig. 2C). The score was designed to prioritize molecules with satisfactory dose-response profile, low EC50 and high specificity, calculated as the fold difference between the EC50s from screening and counterscreening assays. For this screen, the H2LPS ranged from 0 to a maximum of 276.24. Using this approach, 14 of the 27 identified hits had an H2LPS score >0 (Table 2). We selected the 10 highest scoring hits from this subset for *in vitro* validation studies. We treated C_2_C_12_ cells with 1 µM concentrations of the selected hits for 24 hours, followed by western blot evaluation of utrophin levels as an orthogonal assay for endogenous protein expression. Utrophin protein levels, normalized to tubulin, were significantly higher than the 0.1% DMSO control for 8 out of 10 compounds (fold increase > 1.5 and P < 0.05) (Fig. 2D,E). Panobinostat, a non-selective HDAC inhibitor for cancer treatment, increased utrophin levels by 1.9-fold. One of the hits, Givinostat (S2170), a potent pan-HDAC inhibitor with previously demonstrated potential for treating DMD^47,48^, upregulated utrophin by 2.2-fold. AR-42 (S2244), another HDAC inhibitor with proven preclinical efficacy in contrasting cancer-induced cachexia^49^, increased utrophin by 2.3-fold and had the second highest H2LPS. Consistent with the calculated H2LPS, the highest scoring hit, TSA (S1045), had also the highest magnitude of protein upregulation (3.9-fold) followed by PCI-24781 (S1090; 3.7-fold). Importantly, high H2LPS was generally correlated with confirmation in WB assays (Fig. 2E).

**Table 2:** Hit 2 Lead Performance Score (H2LPS).

Rank	Cat. No.	Compound	Cluster	EC50 screen	Fold diff	Curve shape score (0/1)	Upper-lower limits screen	EC50 score (1 to 4)	Fold diff score (0 to 2)	H2LPS*
1	*S1045*	*Trichostatin A (TSA*)	1	0.013	119.82	1	34.53	4	2	276.24
2	*S*2*244*	*AR-42*	4	0.369	14.72	1	59.94	2	2	239.76
3	*S1515*	*Pracinostat (SB939*)	4	0.558	59.25	1	56.37	2	2	225.48
4	*S217*0	*Givinostat (ITF*2*357*)	4	0.269	18.69	1	46.59	2	2	186.36
5	*S1090*	*PCI-24781 (Abexinostat)*	4	0.138	33.72	1	44.18	2	2	176.72
6	*S1085*	*Belinostat (PXD101)*	4	0.342	High	1	43.41	2	2	173.64
7	*S7569*	*LMK-235*	4	0.683	High	1	43.40	2	2	173.6
8	*S8043*	*Scriptaid*	3	2.438	High	1	62.01	1	2	124.02
9	*S1030*	*Panobinostat (LBH589)*	1	0.013	9.21	1	22.06	4	1	88.24
10	*S2779*	*M344*	3	3.218	2.54	1	74.15	1	1	74.15
11	*S1194*	*CUDC-101*	3	1.991	High	1	34.34	1	2	68.68
12	*S7917*	*Kenpaullone*	6	0.034	6.97	1	12.92	4	1	51.68
13	*S7597*	*BV-6*	6	0.099	High	1	7.00	3	2	42
14	*S7198*	*BIO*	6	0.125	3.01	1	12	2	1	24
15	*S7648*	*OTS964*	1			0		4	0	0
16	*S1095*	*LAQ824 (Dacinostat)*	1			0		4	0	0
17	*S1096*	*Quisinostat (JNJ-26481585)*	1			0		4	0	0
18	*S3020*	*Romidepsin (FK228, Depsipeptide)*	1			0		4	0	0
19	*S3030*	*Niclosamide (Niclocide)*	6			0		4	0	0
20	*S7652*	*OTS514 hydrochloride*	2			0		4	0	0
21	*S2349*	*Rutaecarpine*	6			0		4	0	0
22	*S2892*	*VU 0361737*	6			0		4	0	0
23	*S2422*	*Ipriflavone (Osteofix)*	6			0		4	0	0
24	*S2759*	*CUDC-907*	5			1		4	0	0
25	*S7665*	*CH5183284 (Debio-1347)*	6			0		4	0	0
26	*S8376*	*Rbin-1*	6			0		4	0	0
27	*S7915*	*BIO-acetoxime*	6	0.33	0.65	1	13.04	2	0	0

*H2LPS = Curve Shape Score * (Upper limit − Lower Limit) * EC50score * Fold Diff Score.

### *In vitro* validation of the highest scoring hit, TSA

**Figure 3:**
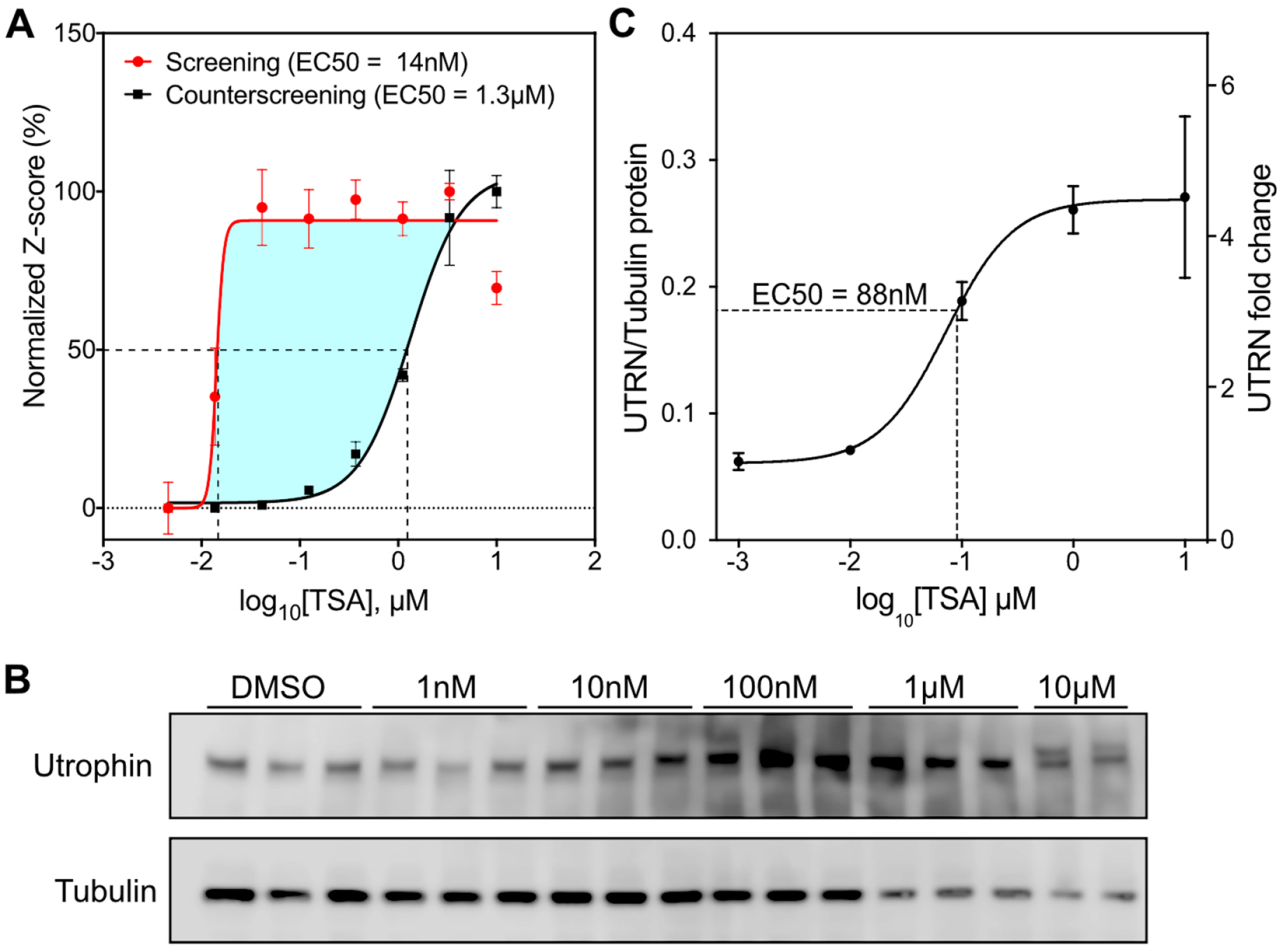
*In vitro* validation of the highest scoring hit (TSA – S1045). (**A**) Dose-response traces of Z-scores for S1045 on the screening and counterscreening assays. For comparison purposes, both traces are normalized to 100%, without affecting their EC50. (**B**) Utrophin western-blot of C_2_C_12_ treated for 24 hours with increasing concentrations of TSA, and quantification (**C**) fitted with dr4pl to calculate the EC50 for the utrophin protein levels. Values are mean and standard error of the mean.

TSA is a broad-spectrum HDAC inhibitor and its therapeutic potential for DMD was previously suggested^50,51^. One of the factors contributing to its high H2LPS is the significant window between the screening EC50 (13 nM) and counterscreening EC50 (1.3 µM) (Fig. 3A). To further validate *in vitro* the dose-dependent capacity of TSA to upregulate utrophin protein, we treated C_2_C_12_ cells for 24 hours with increasing concentrations of TSA (from 1 nM to 10 µM) (Fig. 3B). Consistent with the single-dose western blot validation experiments, utrophin protein levels were increased 4.3 times by 1 µM TSA. The calculated EC50 for utrophin protein levels was 88 nM (Fig. 3C). Some cytotoxicity was evident for concentrations of TSA higher than 1 µM. This data suggests that, at nanomolar concentrations of TSA, the interaction with the 5′ and/or 3′UTRs of the utrophin significantly contributes to the increase in utrophin expression.

### *In vivo* TSA treatment in the mdx mouse model of DMD

**Figure 4:**
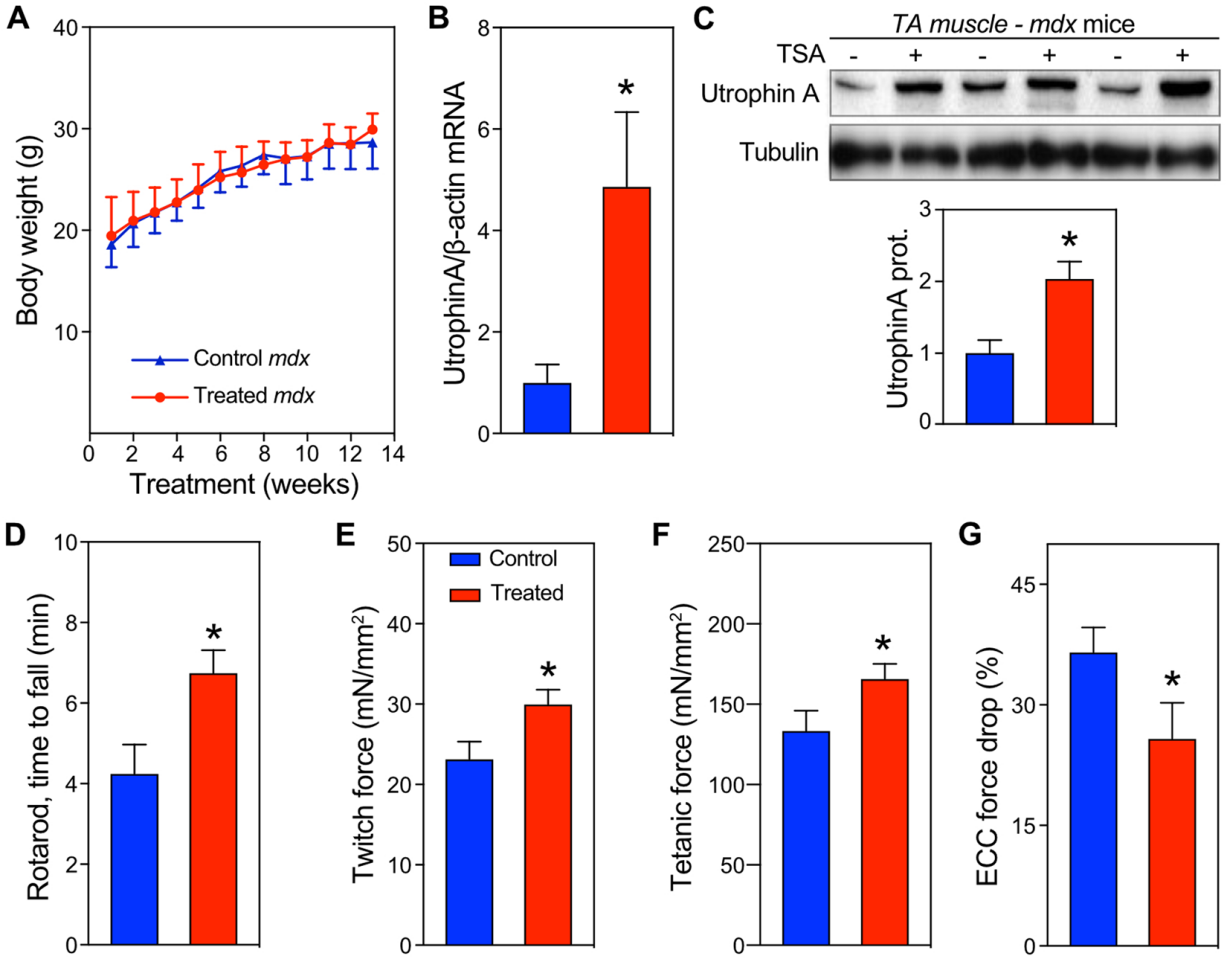
*In vivo* preclinical validation of TSA treatment in the *mdx* mouse model of DMD. (**A**) Body weight monitored during TSA treatment [n = 12/group]. Utrophin mRNA (**B**) [n = 5 ctrl and 6 treated] and protein (**C**) [n = 3/group] levels in TA muscle after TSA treatment. (**D**) Rotarod performance test after TSA treatment [n = 12/group]. Twitch (**E**) [n = 18 muscles/group] and tetanic (**F**) [n = 18 muscles/group] specific forces of EDL muscles after TSA treatment. (**G**) EDL force decrement after 5 consecutive eccentric contractions. Values are mean and standard error of the mean. *p < 0.05.

To test the ability of TSA to increase utrophin *in vivo* and improve the dystrophic phenotype, we treated 4-week-old *mdx* mice with i.p. injections of 30 µg/kg body weight TSA, on alternating days for a total of 3 months. TSA did not affect body weight (Fig. 4A) or the wet weight of skeletal muscles and organs compared to control-treated mice (Table 3). Utrophin mRNA (Fig. 4B) and protein (Fig. 4C) levels were increased by 4.9-fold and 2-fold respectively in TA muscle. Immunofluorescence staining showed increased utrophin expression at the sarcolemma and at the neuromuscular junction (Suppl. Fig. 3). The higher utrophin expression was associated with a significant increase in muscle performance both *in vivo* (Fig. 4D) and *ex vivo* (Fig. 4E,F). Treated *mdx* mice performed significantly better than control *mdx* on a rotarod test, which assays motor coordination, postural control and fatigability^52^ (Fig. 4D). Consistently, isometric twitch and tetanic strength of EDL muscles were significantly higher in TSA treated mice, both before (Table 4) and after normalization by crossectional area (Fig. 4E,F and Table 4), suggesting that TSA improved muscle contractility without increasing muscle mass. In addition, at the dosage used, TSA treatment did not cause changes in myostatin or follistatin gene expression (Suppl. Fig. 4). No significant changes were noted in serum creatine kinase or hydroxyproline content in TA and diaphragm muscles (Table 4). A key parameter to evaluate when testing treatments for DMD is susceptibility of muscle to damage caused by repeated eccentric muscle contractions (ECC)^52–54^. By applying a series of 5 ECCs, we found that TSA treatment protected *mdx* muscles from eccentric damage, as they lost on average 37% less force than control-treated muscles after the 5^th^ ECC (Fig. 4G). Consistently, procion orange uptake was significantly lower in treated muscles compared to untreated (Suppl. Fig. 5). These improvements of both *in vivo* and *ex vivo* parameters were accompanied by a 30% lower number of centrally nucleated fibers (Table 4) in TSA-treated EDL muscles. Together, these data suggest that TSA treatment significantly improved the dystrophic phenotype in the *mdx* mouse model of DMD.

**Table 3:** Body, muscle and organ weights of control and treated *mdx* mice.

	n	Control *mdx*	Treated *mdx*	*P*
Body weight (g)	11 (mice)	29.2 ± 0.8	30.2 ± 0.5	0.3008
Muscles (mg)	18 (muscles)			
EDL		17.6 ± 0.6	17.5 ± 0.4	0.5785
Soleus		13.2 ± 0.7	12.8 ± 0.4	0.6088
Tibialis Anterior (TA)		68.0 ± 2.2	66.1 ± 1.5	0.4855
Gastrocnemius		194.6 ± 6.3	213.0 ± 11.0	0.5062
Quadriceps		239.9 ± 10.9	234.6 ± 8.7	0.7058
Organs (mg)	11 (organs)			
Heart		170.6 ± 10.9	159.7 ± 5.2	0.5503
Liver		1260.0 ± 85.0	1179.0 ± 36.4	0.6063
Kidneys	8	544.3 ± 18.8	532.3 ± 25.8	0.7106

**Table 4:** Contractile, morphometric and biochemistry measurements.

	n	Control *mdx*	Treated *mdx*	*P*
*EDL contractility*				
Twitch force (mN)	18	49.91 ± 4.61	64.19 ± 4.22	0.0287*
Twitch force (mN/mm^2^)	18	23.19 ± 2.15	29.98 ± 1.84	0.0220*
Tetanic force (mN)	18	284.6 ± 23.42	351.9 ± 16.61	0.0250*
Tetanic force (mN/mm^2^)	18	133.5 ± 12.50	166.0 ± 9.09	0.0434*
ECC force drop 1-5 (%)	18	36.58 ± 3.09	25.81 ± 4.46	0.0114*
*EDL morphometry*				
L_0_ (mm)	18	11.34 ± 0.20	12.16 ± 0.20	0.0068**
CSA (mm^2^)	18	2.18 ± 0.09	2.20 ± 0.13	0.8717
CNF (%)	18	34.82 ± 2.72	24.62 ± 2.30	0.007**
*Biochemistry*				
Serum CK (U/L)	3 vs 4	10492 ± 4104	7450 ± 2381	0.6286
Diaphragm Hydroxyproline TA (µg/mg)	6	0.92 ± 0.02	0.90 ± 0.06	0.2814
Diaphragm Hydroxyproline Diaphragm (µg/mg)	6	3.44 ± 0.22	3.33 ± 0.22	0.4156

CSA, cross sectional area; ECC, eccentric contraction; L_0_, muscle length; CNF, centrally nucleated fibers.

## Discussion

In this study, we performed an HTS to identify post-transcriptional up-regulators of utrophin expression. We scaled our previously described assay to a 384-well format, however, the technical parameters of the assay (Table 1 and Suppl. Fig. 1A–C) suggest that the assay is amenable to scaling-up to higher throughput formats (e.g. 1536-well) that would allow additional, more diverse libraries to be screened. Since our goal for this HTS was to validate our strategy and identify small molecules capable of increasing utrophin expression with a view to DMD therapeutics, we screened a curated library of 3127 small molecules, enriched in FDA approved drugs and compounds with known pharmacological activity (Fig. 1). Our initial screen identified 27 hits (Fig. 2). By performing dose-response analyses in screening and counterscreening, and prioritizing the hits using our H2LPS algorithm (Fig. 2C), we were able to identify a subset of 14 compounds that are highly likely to increase utrophin by targeting the mechanisms of post-transcriptional repression. A significant advantage of ranking hits with the H2LPS is that it can be computed automatically, ensuring objective evaluation and applicability to larger screening libraries. Using this approach, we excluded 13 molecules with H2LPS = 0 due to poor dose-response or significant activity in the counterscreen, and validated the 10 highest scoring molecules. Consistent with the high H2LPS, 8 out of 10 molecules significantly increased utrophin protein more than 1.5 times in C_2_C_12_ cells (Fig. 2D,E). In a separate assay, hits with score equal to 0 (e.g. S7652, S1095, S1096 and S3020) failed to increase utrophin protein levels (data not shown), supporting the predictive value of the H2LPS as an efficient algorithm to optimize the progression of hits to leads for drug development.

The development of pharmacological strategies to upregulate utrophin offers a number of translational advantages over conventional approaches for dystrophin-replacement using gene therapy or stem cells. Since utrophin expression is unabated in DMD patients, our small molecule approach to upregulate utrophin should circumvent many of the hurdles associated with delivery, toxicity and immune reaction of conventional DMD gene therapy (e.g. immune reactions against dystrophin itself or the capsid components of the viral vectors used for gene therapy). In addition, while the cloning capacity of the currently available viral vectors requires the use of smaller, internally truncated forms of dystrophin or utrophin, our approach allows us to target the natural, full length version. The use of small molecules offers advantages in terms of delivery, stability and bioavailability, especially in case of drugs that already exist in the pharmacopeia (e.g. repurposing), which already have been optimized for absorption, distribution, metabolism and excretion and toxicity (ADMET) properties in humans and are a prime focus of ongoing experiments in our laboratory.

Our screening independently highlights the potential of two molecules (Givinostat and TSA) that have been previously suggested for DMD therapy. Givinostat (S2170) was recently shown to improve DMD pathophysiology by reducing inflammatory infiltration and fibrosis, and promoting muscle regeneration in *mdx* mice and in boys with Duchenne^47,48^ and is currently in Phase III trials in humans. TSA (S1045), has previously been shown to have pro-myogenic effects in C_2_C_12_ cells^55–58^, protect against unloading-induced muscle atrophy^59^, increase tetanic force in dystrophic myotubes^50^, and improved the dystrophic phenotype in *mdx* mice^51^. Previous studies have shown that TSA can directly activate the utrophin promoter^33^ as well as increase follistatin-mediated muscle regeneration^51^. Here we demonstrate that TSA can also increase utrophin levels post-transcriptionally by interacting with the 5′ and/or 3′UTR in the utrophin mRNA. In our assays the EC50 for TSA was 100 times lower when the reporter transgene carries the 5′3′UTR (screening assay) compared to when only the CMV promoter was present (counterscreening assay). We suggest that the mechanism(s) of action by which TSA increases utrophin expression are likely multiple and dose-dependent, acting on utrophin promoter and UTRs. A number of our hits (Givinostat, TSA and AR-42) are known HDAC inhibitors and hence are likely to demonstrate broad ranging effects in cellular and *in vivo* assays. Future studies will focus on separating the HDAC activity from the utrophin promoting activity to increase specificity and efficacy. Indeed, TSA is commonly used to inhibit HDAC activity and proliferation of cancer cells, with IC50 *in vitro* in the nM to µM range, depending on the particular cell line^60^. *In vivo*, doses up to 5 mg/kg or 10 mg/kg have been used to exert anti-tumor activity^60^ and prevent motor neuron death in a model of spinal muscular atrophy^61^, respectively. In this study, we treated *mdx* mice with 30 µg/kg TSA (~1 µM for a 20 g mouse) on alternating days for 14 weeks (Fig. 4). Using this paradigm, utrophin upregulation was independent of changes in myostatin or follistatin (Suppl. Fig. 4). The dosage used in our study is lower than what is generally used *in vivo* to inhibit HDAC activity and tumor progression^62^. Therefore, higher dosages or treatment frequency, or combinatorial drug strategies, may enhance the benefits.

In conclusion, we used an HTS-based strategy to identify and validate utrophin post-transcriptional up-regulators. As a validation of the approach, preclinical testing of our top scoring lead molecule (TSA) confirmed that it significantly improves muscle structure and function in the *mdx* mouse model of DMD. The hits identified here may be complementary to those acting on the utrophin promoter as well as other strategies such as gene therapy or exon skipping. Such combinatorial approaches may help potentiate the benefits of these molecules for DMD therapy. Taken together, we believe our screening and counterscreening strategy, together with H2LPS ranking will significantly facilitate future screenings of larger libraries as well. Additional preclinical studies and clinical trials on the molecules identified here, along with additional HTS efforts which are currently underway in our laboratory, should help efficiently harness the benefits of this approach for DMD.

## Methods

### Screening and counterscreening cell lines and HTS implementation

The construct for the HTS assay was generated by cloning the 5′- and 3′-UTRs of human utrophin into pGL4:50-hygro (Promega, Madison, WI) at the 5′ and 3′ of the luciferase coding sequence and has been described previously by our group^46^. For the counterscreening assay, we used the pGL4:50-hygro plasmid alone. Low passage C_2_C_12_ cells obtained from ATCC were transfected with either constructs, stably selected with 500 µg/ml hygromycin and subcloned using two rounds of serial dilutions to isolate single cell-derived colonies in a 96 wells plate format. Stable clones were tested to verify the presence of the transgene, luciferase activity and absence of mycoplasma. The selected clones were cultured in presence of 100 µg/ml hygromycin B and were transferred to the HTS Facility at the University of Pennsylvania for implementation into a 384-wells format suitable for HTS. For all the screening and validation experiments, cells were plated at a density of 20000 cells/cm^2^ and maintained in DMEM-based standard proliferation medium. Care was taken to avoid conditions favoring cell fusion or differentiation. Technical data for the screening lines are reported in Table 1.

### Screening library

We screened a custom generated library of small molecules enriched for FDA approved compounds (1164) and compounds with known pharmacological activity (1836) from Selleckchem. The library consists of 373 known kinase inhibitors, 246 compounds classified as cancer chemotherapeutics, 150 inhibitors of epigenetic regulators, 358 GPCR and Ion Channel modulators, with the remaining 2000 compounds falling into diverse target classes (e.g. protease inhibitors, anti-infectives, etc.). Compounds were suspended in DMSO, arrayed in columns 3–22 of 384 well microplates, and stored at −20 °C. Library plates were thawed a maximum of 10 times to maintain compound integrity.

### High Throughput screening

We seeded 1000 cells in a volume of 25 μl per well of 384-well Corning 3750 microplates using a Multidrop^TM^ Combi Reagent Dispenser (Thermo Scientific). Cells were allowed to attach overnight at 37 °C, 5%CO_2_ in a humidified chamber. Compounds (50nL) were transferred to assay plates using a 384, 50nL slotted pin tool (V&P Scientific) and a JANUS Automated Workstation (Perkin Elmer). Compounds were added to a final concentration of 1uM and 100 nM in 0.2%DMSO. Columns 1 and 23 were treated with 0.2%DMSO (negative control). Columns 2 and 24 were treated with 100 nM Panobinostat (positive control). Cells were incubated for 24 hours at 37 °C, 5% CO_2_. Assay plates were removed from the incubator for 1 hour to equilibrate to room temperature, prior to adding 25 μL of 0.5X Britelite (PerkinElmer). Luminescence was measured on an EnVisionXcite Multilabel Plate Reader (PerkinElmer), using ultrasensitive luminescence measurement technology.

### Data Analysis and H2LPS score

Raw values from DMSO and Panobinostat control wells were aggregated and used to calculate z′-factors [1 − (3*(DMSO_sd_ + Panobinostat_sd_)/Abs(DMSO_avg_ − Panobinostat_avg_)] for each assay plate, as a measure of assay performance and data quality, with a z’-factor > 0.5 representing acceptable data. Raw data values of sample wells were normalized to aggregate DMSO and Panobinostat plate control wells and expressed as Normalized Percent Activation [NPAc = ((DMSO_avg_ − Test well)/(DMSO_avg_ − Panobinostat_avg_)) × 100] and Z-score [Z = (Test well − DMSO_avg_)/(DMSO_sd_)]. A hit-rate cutoff of 22.07% activation was calculated as (3*Stdev[NPAc]) + Avg[NPAc]. Data wrangling and visualization were performed in R version 3.5.1. Dose-response analysis was done with the dr4pl package (version 1.1.7.5) for R, using the following parameters: trend = increasing; method.init = logistic; method.robust = Huber.

We designed and developed a Hit 2 Lead Prioritization Score (H2LPS) as a algorithm for ranking and prioritizing hits prior to *in vitro* validation and preclinical evaluation. H2LPS is a unitless parameter designed to be directly proportional to the performance of the hit on the screening assay using multiple parameters. To obtain the H2LPS, initial scores were calculated separately for EC50_screen_, fold difference between the EC50s of screening and counterscreening, and success in fitting a 4-parameters logistic dose-response curve using the dr4pl package for R. A Curve Fitting score of 1 was given if the dose-response data from the screening could be successfully fitted using the dr4pl package, or 0 if the fitting was not successful. The difference between upper and lower boundaries of the fitted dose-response screening curve was factored in the final H2LPS score calculation. The EC50_screen_ score was 4 if EC50_screen_ < 50 nM (or if no EC50 could be calculated), 3 if EC50_screen_ < 100 nM, 2 if EC50_screen_ < 1 µM and 1 if EC50_screen_ > 1 µM. Finally, the Fold Difference score was 2 if fold difference > 10, 1 if fold difference > 2 and 0 if fold difference ≤ 2. The final formula for the H2LPS therefore was H2LPS = Curve Fitting score *(upper – lower limits of fit) *EC50_screen_ score * Fold Difference score. For ease of presentation in the manuscript, the upper-lower limit values were rounded to the second decimal place.

### Preclinical studies in *mdx* mice

All animal experiments were conducted in accordance with protocols approved by the Institutional Animal Care and Use Committee of the University of Pennsylvania. Mice were housed at 22 °C under a 12:12-h light-dark cycle with food and water provided ad libitum. Four-week-old *mdx* mice (C57BL/10ScSn-DMDmdx/J) were injected intraperitoneally on alternate days with Trichostatin A (Wako Chemicals USA-Inc, Richmond, VA, USA; dose 30 µg/kg), or an equal volume of sterile PBS for three months.

Functional *in vivo* and *ex vivo* analyses including rotarod, EDL strength, and sensitivity to damage induced by lengthening contractions (ECC), were performed as previously described^63,64^. Following the procedure, muscles were flash-frozen in liquid nitrogen-cooled isopentane and stored at −80 °C.

### Gene expression and western blot analyses

Total RNA was extracted from TA muscle samples with RNAeasy kit (Qiagen, USA) and reverse transcribed with random hexamers. qPCR was performed using Taqman probes for Utrophin (Assay ID Mm01168846_m1) and β-actin. Muscle tissue samples were lysed with TNEC buffer (50 mM Tris-HCl, pH 8.0; 150 mMNaCl; 1% NP40; 2 mM EDTA) containing complete protease inhibitor cocktail (Roche, Basel, Switzerland). C_2_C_12_ cells were treated for 24 hours with 1 µM concentrations of compounds from a fresh batch (3 wells for each compound), and cell samples were lysed with RIPA buffer with protease inhibitor cocktail. The experiment was repeated three independent times. 50–75 µg of total proteins from muscle tissues, or 4 µg of cell protein extracts, were resolved on a 3–8% Tris-Acetate gradient gel (NuPage; Invitrogen), transferred to PDVF or nitrocellulose membranes and mouse monoclonal anti-utrophin antibody MANCHO3 clone 8A4 (developed by Glenn E. Morris and obtained from the Developmental Studies Hybridoma Bank, Iowa) or a custom-made anti-UtrophinA rabbit polyclonal antibody (muscle tissue samples) generated by us and described previously^28^.

### Statistical analysis

Values are presented as mean ± standard error of the mean. Data visualization and statistical analysis for the *in vivo* studies was performed using GraphPad Prism8. Comparisons between two groups were done using a two-tailed Student t-test or Mann-Whitney non-parametric test. For the western blot validation experiments, in order to account for the variability between experiments and between gels, data were analyzed in R using a linear mixed model. All statistical tests were considered significant at a α ≤ 0.05 unless stated otherwise.

## Supplementary information

Supplementary Information.
