# Author Correction: High-throughput identification of post-transcriptional utrophin up-regulators for Duchenne muscle dystrophy (DMD) therapy

**DOI:** 10.1038/s41598-020-60885-8

**Published:** 2020-02-28

**Authors:** Emanuele Loro, Kasturi Sengupta, Sasha Bogdanovich, Kanupriya Whig, David C. Schultz, Donna M. Huryn, Tejvir S. Khurana

**Affiliations:** 1 Department of Physiology and Pennsylvania Muscle Institute, Perelman School of Medicine, University of Pennsylvania, Philadelphia, PA USA; 2 High-Throughput Screening Core, University of Pennsylvania, Philadelphia, PA USA; 3 Department of Pharmaceutical Sciences, University of Pittsburgh, Pittsburgh, PA USA

Correction to: *Scientific Reports* 10.1038/s41598-020-58737-6, published online 07 February 2020

The original version of this Article contained a typographical error in the Abstract.

“We identified 27 hits that were ranked using a using an algorithm that we designed for hit prioritization that we call Hit to Lead Prioritization Score (H2LPS).”

now reads:

“We identified 27 hits that were ranked using an algorithm that we designed for hit prioritization that we call Hit to Lead Prioritization Score (H2LPS).”

This has now been corrected in the PDF and HTML versions of the Article.

