# Supplementary material for: High-throughput identification of post-transcriptional utrophin up-regulators for Duchenne muscle dystrophy (DMD) therapy
Source: Sci Rep. 2020 Feb 7;10:2132. doi: 10.1038/s41598-020-58737-6 (PMC7005813; doi:10.1038/s41598-020-58737-6)
Supplement: Supplementary file 1 — Supplementary Information. [file 41598_2020_58737_MOESM1_ESM.pdf]

Supplementary Information

High-throughput identification of post-transcriptional utrophin up-regulators for Duchenne muscle dystrophy (DMD) therapy.

Emanuele Loro[1], Kasturi Sengupta[1], Sasha Bogdanovich[1], Kanupriya Whig[2], David Schultz[2], Donna M. Huryn[3], Tejvir S. Khurana[1*].

[1]Department of Physiology and Pennsylvania Muscle Institute, Perelman School of Medicine, University of Pennsylvania, Philadelphia, PA, USA.

[2]High-Throughput Screening Core, University of Pennsylvania, Philadelphia, PA, USA.

[3]Department of Pharmaceutical Sciences, University of Pittsburgh, Pittsburgh, PA, USA.

Supplementary methods

Immunofluorescence and histochemical labeling of muscles.

10μm frozen sections of tibialis anterior muscles were stained using a 1 to 100 dilution of rabbit polyclonal antibody against utrophin A generated by us and described previously[1]. Neuromuscular junctions were labeled with bungarotoxin Alexa Fluor 594 conjugated.

For procion orange uptake, EDL muscles were incubated in a 1% solution of procion orange dye in ringer buffer for 15 minutes, before proceeding with standard freezing procedures using tissue freezing medium and isopentane cooled in liquid nitrogen. 10μm sections were then cut and imaged.

Stained sections were imaged using an Olympus BX51 microscope equipped with an Olympus DP12 digital camera.

Supplementary bibliography

Baby, S. M. *et al.* Differential expression of utrophin-A and -B promoters in the central nervous system (CNS) of normal and dystrophic mdx mice. *Brain Pathol.* **20,** 323–342 (2010).

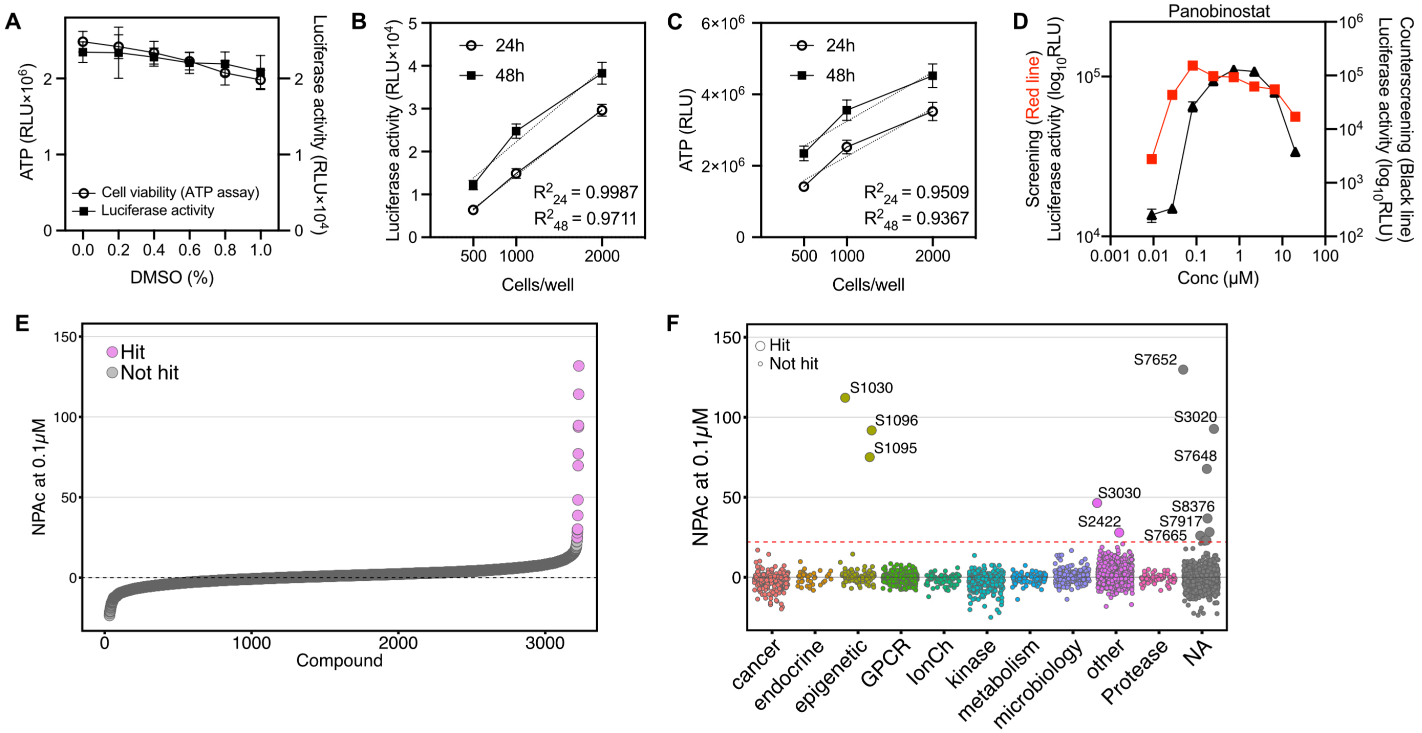

Supplementary Figure 1: Technical validation of the high-throughput screening cell line. (**A**) Cell viability (ATP) and luciferase activity following 24 hours incubation with increasing concentrations of DMSO. (**B**) Luciferase activity and cell viability (**C**) at different plating densities and incubation times. Linear fit demonstrated the linearity of response as a function of cell density. (**D**) Panobinostat dose-response using the screening and counterscreening assays. (**E**) Scatter plot of Normalized Percent Activation (NPAc) for each compound, assayed at 0.1μM concentrations. (**F**) Scatter plot of NPAc for the different compounds assayed at 0.1μM concentrations, organized into different target classes. Values are mean and standard error of the mean.

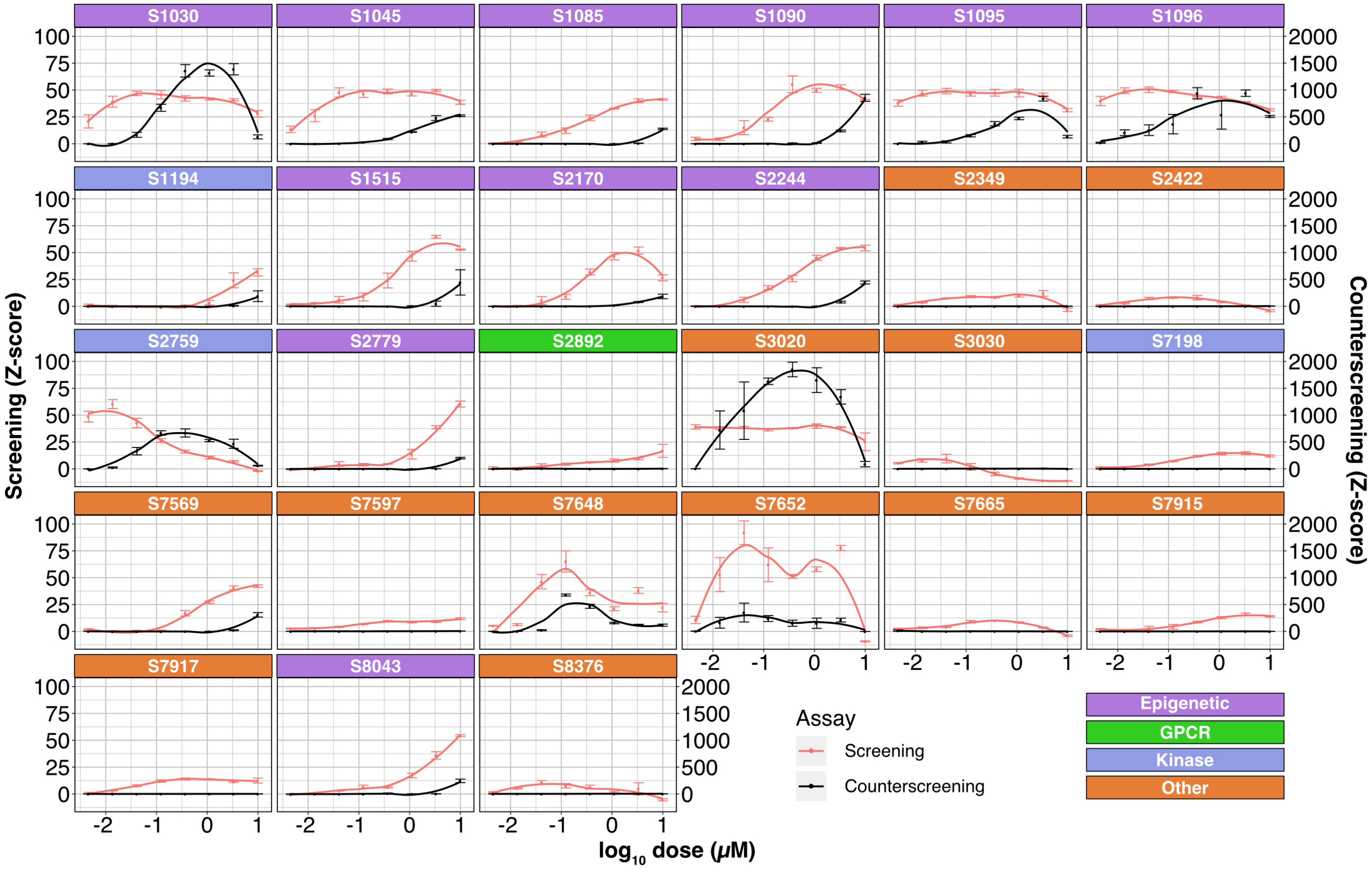

Supplementary Figure 2: Raw dose-response traces for screening (red) and counterscreening (black) of the 27 selected hits. Values are mean and standard error of the mean [n=4wells/compound]. Headers of different colors identify the target class.

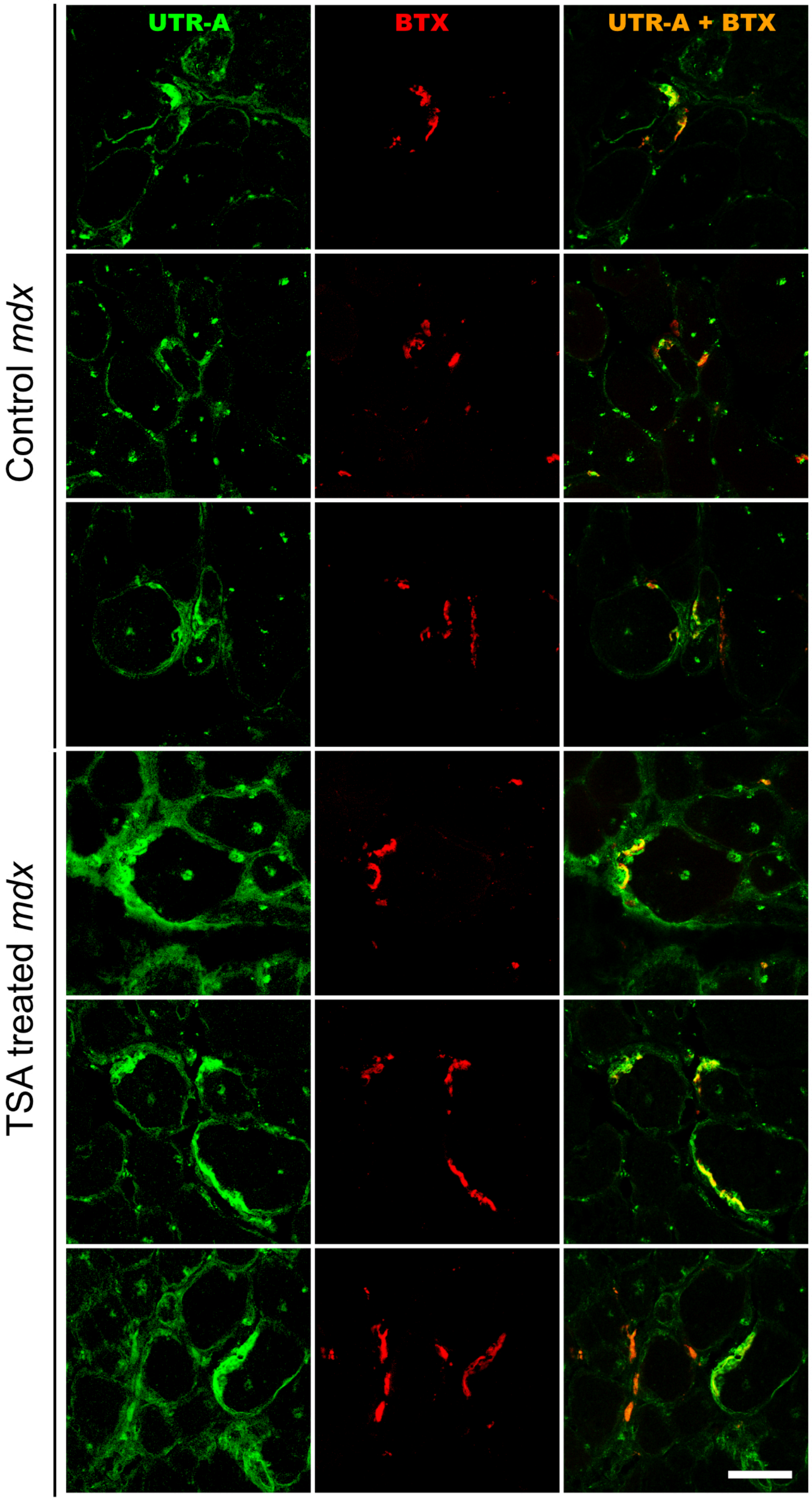

Supplementary Figure 3: Representative immunofluorescence images of utrophin A in control and TSA-treated tibialis anterior muscle. Bungarotoxin staining (red) was used to label neuromuscular junctions. Scalebar 50μm.

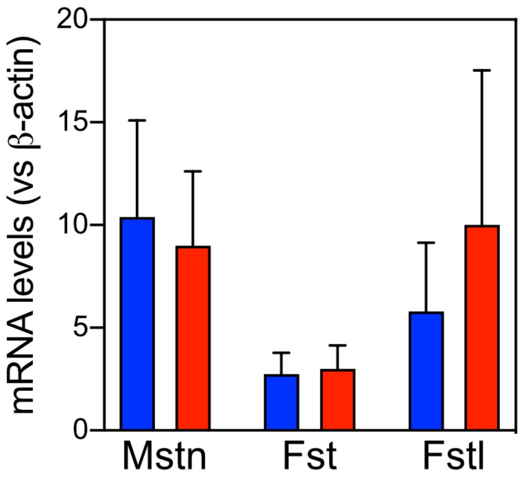

Supplementary Figure 4: qPCR analyses of TSA-treated muscles. mRNA levels of myostatin, follistatin and follistatin-related protein in TA muscles of *mdx* mice following TSA treatment [n=5/group].

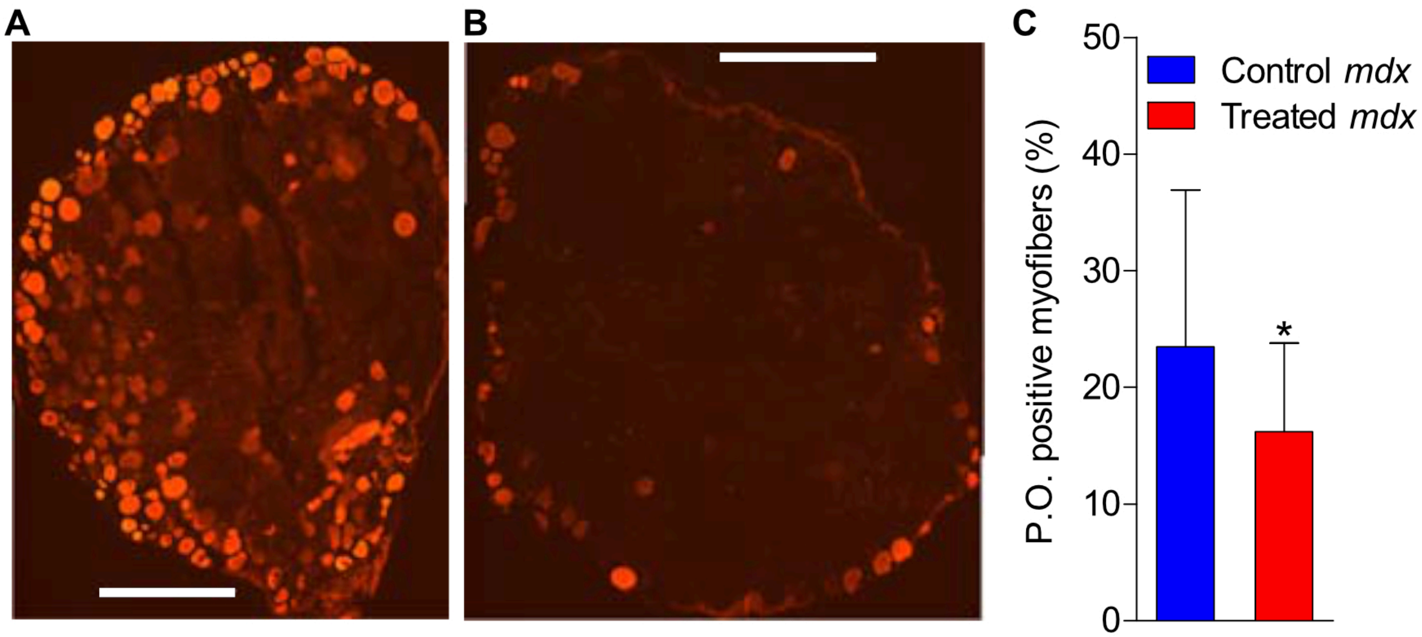

Supplementary Figure 5: Procion orange uptake in control and TSA-treated EDL muscles.
Representative images (**A,B**) with quantification (**C**) of positive myofibers per section [n=18/group]. Scalebar 200μm.
